# Newly designed liquid chromatographic method using relative molar sensitivity based on ^1^H-qNMR for quantifying polymethoxyflavones from *Kaempferia parviflora*

**DOI:** 10.1016/j.fochx.2026.104115

**Published:** 2026-06-18

**Authors:** Daigo Iwasaki, Hiroaki Kawamoto, Toshiyuki Murakami, Takashi Ohtsuki, Hiroshi Matsufuji

**Affiliations:** a Graduate School of Bioresource Sciences, Nihon University, 1866, Kameino, Fujisawa-City, Kanagawa 252-0880, Japan; b Research Center, Maruzen Pharmaceuticals, Co., Ltd., 1089-8, Sagata, Shinnichi-Cho, Fukuyama-City, Hiroshima 729-3102, Japan

**Keywords:** *Kaempferia parviflora*, Polymethoxyflavones, Single-reference method, Relative molar sensitivity, ^1^H-qNMR, UHPLC, 5,7,3′,4′-Tetramethoxyflavone (PubChem CID: 631170), 3,5,7,3′,4′-pentamethoxyflavone (PubChem CID: 97332), 5,7-dimethoxyflavone (PubChem CID: 88881), 5,7,4′-trimethoxyflavone (PubChem CID: 79730), 3,5,7-trimethoxyflavone (PubChem CID: 117900), 3,5,7,4′-tetramethoxyflavone (PubChem CID: 631095)

## Abstract

Quality control of six polymethoxyflavones in *Kaempferia parviflora* (KP) extract, a functional food widely used in Japan, is limited by the availability of high-purity reference standards. To address this, we developed a new analytical method with relative molar sensitivity based on ^1^H-quantitative nuclear magnetic resonance spectroscopy. This approach enables simultaneous quantification of six polymethoxyflavones using 5,7-dimethoxyflavone as a single-reference standard, combined with ultrahigh-performance liquid chromatography employing a superficially porous particle column and a ternary mobile phase (detection at 265 nm). The method achieved a limit of quantification of 2.00 μmol/L, and specificity was confirmed by comparison with liquid chromatography–tandem mass spectrometry. Accuracy and precision met the acceptance criteria established by AOAC guidelines. By reducing reliance on multiple standards, this method provides a practical solution for the quality assurance of KP-derived products. Its validation, aligned with internationally recognized frameworks, further supports its potential for application in other regions.

## 1 Introduction

The rhizome of *Kaempferia parviflora* (KP), a perennial herb in the Zingiberaceae family, is consumed as food. It contains six principal polymethoxyflavones (PMFs) that have recently attracted attention for their functional properties. These PMFs are (i) 5,7,3′,4′-tetramethoxyflavone (PMF1), (ii) 3,5,7,3′,4′-pentamethoxyflavone (PMF2), (iii) 5,7-dimethoxyflavone (PMF3), (iv) 5,7,4′-trimethoxyflavone (PMF4), (v) 3,5,7-trimethoxyflavone (PMF5), and (vi) 3,5,7,4′-tetramethoxyflavone (PMF6) (Mohammad Aidiel et al., 2024).

Previous studies have reported that KP extracts and their constituent PMFs exhibit anti-obesity effects, physical performance enhancement, and antioxidant properties (Na Takuathung et al., 2024). These effects are supported by studies demonstrating enhanced metabolic activity in brown adipose tissue (Yoshino et al., 2014), significant reductions in abdominal fat (Yoshino et al., 2018), improvements in muscle metabolism and endurance, and activation of antioxidant genes that directly support improved exercise capacity (Huang, Tagawa, Ma and Suzuki, 2022, Huang et al., 2024). Obesity and age-related declines in physical function are major public health concerns. Accordingly, functional ingredients such as KP and its PMFs are attracting increasing attention for their potential to promote metabolic health and physical resilience (Mohammad Aidiel et al., 2024; Na Takuathung et al., 2024).

In Japan, KP extracts are used as ingredients in various functional foods and dietary supplements under the Foods with Function Claims (FFC) framework, which requires standardization of PMF contents of KP extracts. Because the FFC system operates on a notification basis, notifiers are expected to disclose analytical procedures, including information on the purity of reference standards used for quantification, to ensure transparency (Consumer Affairs Agency, 2015; Sakai et al., 2020). Quantification of PMFs in KP extracts typically requires suitable reference materials for each of the six PMFs. However, procurement of natural compound standards is often constrained by limited availability and high cost, as noted in the context of food and drug analysis (USP Council of Experts et al., 2012). In particular, some PMFs in KP extracts are difficult and expensive to obtain owing to a limited number of suppliers.

Several studies have proposed high-performance liquid chromatography (HPLC)-based methods for quantifying PMFs in KP with well-validated precision and accuracy. These studies have contributed substantially to the quality assessment, functional evaluation, and chemotaxonomic differentiation of KP extracts (Asamenew et al., 2019; Joothamongkhon et al., 2022; Krongrawa et al., 2022; Ninomiya et al., 2016). However, these studies were not conducted under conditions that ensured verified purity of the PMF reference standards, highlighting the need for analytical protocols that enable accurate content control based on verified standard purities.

A quantification procedure based on relative molar sensitivity (RMS) has emerged as a practical solution to these challenges. RMS is defined as the ratio of detector response per mole of analyte under a given set of chromatographic conditions (Nishizaki et al., 2020b). For RMS determination, the amount (in moles) of an analyte is typically established by ^1^H-quantitative nuclear magnetic resonance (^1^H-qNMR) spectroscopy. Because RMS expresses detector response on a molar basis, the detector response of a reference standard can be adjusted to that of each analyte using their corresponding RMS values. Consequently, multiple analytes can be quantified using a single-reference standard. This approach has been incorporated into regulatory frameworks such as the Japanese Pharmacopeia and Japan's Specifications and Standards for Food Additives. RMS-based methods have been applied across diverse areas, including crude drugs, foods, food additives, functional foods, and therapeutic drug monitoring, enabling enhanced analytical accuracy and regulatory compliance (Masumoto et al., 2021, Masumoto et al., 2025; Mizumoto et al., 2019; Nishizaki et al., 2018, Nishizaki et al., 2019; Nishizaki et al., 2020a; Ohtsuki et al., 2020, 2024). In our previous study, we proposed an RMS-based quantification method for six khellactone esters in *Peucedanum japonicum* root extracts (Iwasaki et al., 2023) and demonstrated the feasibility of applying this methodology to structurally related natural compounds. In 2025, the guideline “General Requirements for Quantitative Methods Using Relative Molar Sensitivity in Food” was established as a Japanese Agricultural Standard (JAS) by the Ministry of Agriculture, Forestry and Fisheries of Japan (Ministry of Agriculture, Forestry and Fisheries, 2025). International standardization efforts are currently underway via ISO/DIS 25367, reflecting the growing global interest in RMS methodology (International Organization for Standardization, 2026).

Although the general workflow for RMS-based quantification has been systematized in the JAS guidelines, these primarily define overarching principles rather than detailed procedures. In practice, implementation of an operational RMS method requires compound-specific investigations, including determination of RMS values, optimization of chromatographic conditions, and verification of UV-response consistency. In addition, when applying RMS methodology to food samples, the intrinsic complexity of food matrices necessitates additional case-specific evaluation.

To develop an RMS-based method for PMFs, we integrated RMS-based quantification with ultrahigh-performance liquid chromatography (UHPLC) using a sub-2 μm superficially porous particle column. This configuration was adopted to not only address limitations in the availability and purity of PMF reference standards but also enhance the separation performance for the intrinsically complex matrices encountered in food-based samples. Among the six PMFs, PMF3 was selected as the single-reference standard owing to its commercial availability, high purity, chemical stability, and favorable detector response. Nevertheless, because PMFs differ in their UV absorption characteristics, the suitability of a single detection wavelength (265 nm) required careful evaluation.

In this study, we developed an RMS-based method for the simultaneous and accurate quantification of six PMFs in KP extracts. The method was intended for practical use from fundamental research through quality control, addressing the growing need for validated analytical methods in food science and nutrition.

## 2 Materials and methods

### 2.1 Chemical reagents

PMF3 was purchased from Tokyo Chemical Industry Co., Ltd. (Tokyo, Japan) and used as the single-reference standard in this study. The purity of PMF3 was determined to be 99.2 ± 0.3% using ^1^H-qNMR (Section 3.1). PMF1 was purchased from Thermo Fisher Scientific, Inc. (Waltham, MA, USA). PMF2, PMF5, and PMF6 were obtained from TOKIWA Phytochemical Co., Ltd. (Chiba, Japan). PMF4 was obtained from INDOFINE Chemical Company, Inc. (Hillsborough, NJ, USA). The purities of PMF1, PMF2, PMF4, PMF5, and PMF6 were also determined by ^1^H-qNMR in the same manner as PMF3 and ranged from 95.8% to 99.5% (Section 3.1). The KP extract (Lab. No. AC‑04788), a powdered food ingredient product for functional food applications, was provided by Maruzen Pharmaceuticals Co., Ltd. (Hiroshima, Japan). DMSO‑*d*_6_ (deuteration degree ≥99.9%; Thermo Fisher Scientific, Inc., Waltham, MA, USA) and the DSS‑*d*_6_ certified reference material (CRM; FUJIFILM Wako Pure Chemical Corp., Osaka, Japan; Code No. 044-31671, Lot No. ACL6940, purity: 92.3 ± 0.6%) were used for ^1^H-qNMR measurements. Solvents for liquid chromatography (LC) and LC–tandem mass spectrometry (MS/MS), including H_2_O, methanol (MeOH), and acetonitrile (MeCN), were of LC–MS grade and obtained from Kanto Chemical Co., Inc. (Tokyo, Japan). Formic acid (FA, LC–MS grade) and uracil were purchased from FUJIFILM Wako Pure Chemical Corp. Water (H_2_O) for other operations was purified using an Elix 3 system (Merck KGaA, Darmstadt, Germany). Reagent-grade solvents for sample preparation, including MeOH and dimethyl sulfoxide (DMSO), were acquired from FUJIFILM Wako Pure Chemical Corp.

### 2.2 ^1^H-qNMR measurements of PMFs

^1^H-qNMR measurements of PMFs were performed to determine the molar concentrations and purity of each PMF, using DSS‑*d*_6_ (CRM) as the internal standard. PMF1–6 (10.6–14.9 mg) and DSS‑*d*_6_ (1.7–2.6 mg) were accurately weighed, dissolved in DMSO‑*d*_6_, and brought to a final volume of 5 mL. These ^1^H-qNMR solutions for each PMF (hereafter, solution A) also served as the basis for RMS determination, and serial dilutions prepared from these solutions were used to construct LC calibration curves.

The ^1^H-qNMR operating conditions were as follows: observation sweep width, −15 to 25 ppm; acquisition time, 4.0 s; pulse angle, 90°; relaxation delay, 60 s; number of scans, 8; dummy scans, 2; temperature, 40 °C; spinning, off; and ^13^C decoupling, on. Chemical shifts were referenced to the methyl signal of DSS‑*d*_6_ at *δ* 0.00 ppm. Each signal was manually integrated to assess signal integrity and detect impurities.

The molar concentrations (μmol/L) of PMF1–6 were calculated using Eq. (1).

(1) $$C=\left(\frac{I_{\mathrm{PMF}}}{I_{\mathrm{DSS}}}\times \frac{H_{\mathrm{DSS}}}{H_{\mathrm{PMF}}}\times \frac{M_{\mathrm{PMF}}}{M_{\mathrm{DSS}}}\times \frac{W_{\mathrm{DSS}}}{W_{\mathrm{PMF}}}\right)\times P_{\mathrm{DSS}}\times \frac{10^{6}}{V}$$

where *C* is the molar concentration of PMF (μmol/L), *I* is the integrated signal area, *H* is the number of protons contributing to the signal, *W* is the weight of the compound (mg), *M* is the molecular weight (g/mol), *P* is the certified purity of DSS‑*d*_6_ (%), and *V* is the final solution volume (mL). Subscript PMF refers to the PMF of interest (PMF1–6), and DSS refers to internal standard DSS‑*d*_6_. Factor 10^6^ converts the concentration to micromoles per liter (μmol/L). The molecular weight and purity of DSS‑*d*_6_ used in all calculations were 224.35 g/mol and 92.3%, respectively, as stated on the certificate. The molecular weights of PMF1–6 used in the calculations were as follows: PMF1 and PMF6, 342.34 g/mol; PMF2, 372.37 g/mol; PMF3, 282.29 g/mol; PMF4 and PMF5, 312.32 g/mol. The molar concentration of each PMF was determined by averaging values obtained from selected signals chosen to minimize interference from impurities, including overlapping peaks.

The purity (%) of PMF3 was calculated using Eq. (2).

(2) $$P=\left(\frac{I_{\mathrm{PMF}3}}{I_{\mathrm{DSS}}}\times \frac{H_{\mathrm{DSS}}}{H_{\mathrm{PMF}3}}\times \frac{M_{\mathrm{PMF}3}}{M_{\mathrm{DSS}}}\times \frac{W_{\mathrm{DSS}}}{W_{\mathrm{PMF}3}}\right)\times P_{\mathrm{DSS}}$$

where *P* is the purity of PMF3 (%). The variables are defined above. The purity of PMF3 was determined using three independently prepared solutions. The calculated purity was used as the reference value in subsequent quantification procedures.

NMR spectra were acquired on a Varian NMR System 500 (500 MHz; Agilent Technologies, Santa Clara, CA, USA). The analytes (PMF1–6) and DSS‑*d*_6_ were weighed using an ultra-microbalance (MSE2.7S; Sartorius AG, Göttingen, Germany). All spectra were processed using the VnmrJ software (version 3.2; Agilent Technologies).

### 2.3 Apparatus and chromatographic conditions

Three LC systems (A–C) were used for chromatographic analysis (Table S1). LC systems A and B were ACQUITY UPLC H-Class PLUS instruments (Waters Corp., USA) controlled by MassLynx (v4.2). LC system A employed a quaternary pump and a UV detector, whereas LC system B featured a binary pump and a photodiode array (PDA) detector. LC system C was a 1260 Infinity II Prime LC (Agilent Technologies, USA) equipped with a quaternary pump and a PDA detector and controlled by OpenLab CDS ChemStation Edition (vC.01.10). For RMS determination and all quantitative analyses, 2D data were used, whereas 3D PDA data were used for UV spectral measurements.

Chromatographic separation on all LC systems was performed using a Poroshell 120 EC-C18 column (2.1 mm × 150 mm, 1.9 μm; Agilent Technologies, PN 693675–902) maintained at 40 °C, with the sample compartment set to 30 °C. The mobile phases consisted of (A) H_2_O/MeCN/MeOH/FA (450/50/500/1, v/v/v/v) and (B) MeOH/FA (1000/1, v/v). The flow rate was 0.3 mL/min, and the injection volume was 4.2 μL. UV detection was performed at 265 nm. During method development and specificity testing, the gradient program was as follows: 0–27.0 min, 100% A; 27.1–32.0 min, 10% A; and 32.1–37.0 min, 100% A. These conditions were used for RMS determination, LC–UV quantification, and LC–MS/MS analysis. For accuracy and precision testing, a shortened gradient was employed: 0–17.0 min, 100% A; 17.1–22.0 min, 10% A; and 22.1–27.0 min, 100% A.

Three columns from different manufacturing lots were used during method development and validation to enable lot-to-lot evaluation: two columns were used for RMS determination, LC–UV quantification, and LC–MS/MS analysis, and two columns (including one additional lot) were used for accuracy and precision testing. The lot numbers are provided in Table S2.

LC–MS/MS analysis was performed using LC system A coupled to a Xevo TQ-S micro tandem quadrupole mass spectrometer (Waters Corp.). The system was operated under multiple-reaction-monitoring (MRM) conditions with electrospray ionization in positive-ion mode. The injection volume for LC–MS/MS analysis was 0.1 μL. The MRM transitions and associated MS parameters are summarized in Table S3.

Sample preparation involved the use of a microbalance (XPE26V; Mettler Toledo, Greifensee, Switzerland) for samples weighing <20 mg, a semi-microbalance (XSE205DUV; Mettler Toledo) for samples weighing ≥20 mg, and a vortex mixer (S1; IKA, Staufen, Germany). To remove particulate matter, all solutions were filtered through a 0.2 μm polytetrafluoroethylene (PTFE) membrane (DISMIC®-13HP; Toyo Roshi Kaisha) before LC or LC–MS/MS analysis. Data processing and statistical calculations were performed in Microsoft Excel (Microsoft 365, Microsoft Corp., Redmond, WA, USA), and the results of one-way analysis of variance (ANOVA) were verified using R version 4.5.0 (R Foundation for Statistical Computing, Vienna, Austria).

### 2.4 RMS determination based on PMF3

For RMS determination, serially diluted solutions (C–H) of each PMF were prepared for LC analysis as follows. Solution A for each PMF was prepared in ‑*d*_6_ and analyzed by ^1^H-qNMR, as described in Section 2.2. Solution B was prepared by mixing solution A with DMSO and H_2_O/MeOH (50/50, v/v). To ensure consistent dissolution for LC analysis, all subsequent dilutions were performed using a diluent prepared by combining 15 mL of DMSO with H_2_O/MeOH (50/50, v/v) and adjusting the volume to 100 mL. Using this diluent, solution B was serially diluted to prepare solutions C–H at target concentrations of 120, 96.0, 72.0, 48.0, 24.0, and 1.92 μmol/L, respectively. These six concentrations constituted one calibration set used to construct a calibration curve. Actual concentrations were determined from the ^1^H-qNMR results for solution A. The preparation scheme, including the serial dilution steps, is presented in Fig. S1.

For each PMF, three calibration sets were prepared. Each calibration set was analyzed once on LC systems A, B, and C to obtain peak-area data, yielding nine measurements per PMF (three calibration sets × three LC systems). Calibration curves were constructed by plotting the molar concentrations determined by ^1^H-qNMR on the x-axis and the corresponding peak areas on the y-axis. Linearity over the tested range was confirmed, and the regression lines were constrained to pass through the origin.

The RMS values of each PMF relative to PMF3 were determined using the regression coefficients of the calibration curves, as shown in Eq. (3).

(3) $${\mathrm{rms}}_{1-6}=\frac{\beta_{\mathrm{PMF}1-6}}{\beta_{\mathrm{PMF}3}}$$

where *rms*_1–6_ is the RMS value of PMF1–6 relative to that of PMF3, *β*_PMF1–6_ is the regression coefficient of the calibration curve for PMF1–6, and *β*_PMF3_ is the regression coefficient of the calibration curve for PMF3. The final RMS values were determined by averaging nine measurements.

### 2.5 Preparation of sample solutions for quantification

The KP extract (40.0 mg) was accurately weighed, dissolved in 7.5 mL of DMSO, and supplemented with a small amount of H_2_O/MeOH (50/50, v/v). The mixture was thoroughly vortexed and diluted to 50 mL with H_2_O/MeOH (50/50, v/v) in a volumetric flask. The resulting solution was used as the KP solution.

PMF3 (21–23 mg) was accurately weighed, dissolved in DMSO, and adjusted to 7.50 mmol/L. An aliquot of this solution was mixed with DMSO and H_2_O/MeOH (50/50, v/v) to prepare a final standard solution at 120 μmol/L. The solvent composition matched that used for RMS determination (Section 2.4), with 15 mL of DMSO diluted to 100 mL with H_2_O/MeOH (50/50, v/v). The accurate concentration of this solution was calculated by applying the purity of PMF3 determined by ^1^H-qNMR (Eq. (2) in Section 2.2) to the weighed amount.

### 2.6 PMF quantification by the absolute calibration method using LC–UV

To evaluate the RMS-based approach, the absolute calibration method was performed by LC–UV using the same calibration curves constructed during RMS determination (detection at 265 nm). For each calibration set, three parallel preparations of the KP solution were analyzed once on both LC systems, yielding six measurements per calibration set. A total of 3 calibration sets were prepared, giving 18 measurements (3 calibration sets × 3 preparations × 2 LC systems). Individual PMF concentrations were summed to obtain the total PMF content. Final values were reported as the average of all 18 measurements.

The limit of quantification (LOQ) was confirmed by verifying that each PMF in the lowest-concentration solution (solution H) met the signal-to-noise (S/N) ratio criterion of ≥10, calculated according to USP General Chapter <621> (United States Pharmacopeia, 2024).

### 2.7 Specificity evaluation of chromatographic conditions

Accurate chromatographic quantification of target analytes requires careful assessment of potential interference from impurities or matrix components. In this study, specificity was evaluated by comparing LC–UV and LC–MS/MS results under identical chromatographic conditions.

For each PMF group (PMF1, 3, 5 and PMF2, 4, 6), three independent sets of calibration standards were prepared by mixing solution C, diluting 20-fold, and serially diluting to 6.00–0.0960 μmol/L. The KP solution was also diluted 20-fold in triplicate for LC–MS/MS analysis. Quantification was performed in the MRM mode. Calibration curves were constructed by linear regression with 1/x weighting, without forcing the intercept through the origin. Each set was analyzed in duplicate, resulting in six measurements per PMF group. The final PMF contents were calculated as the average of 18 measurements derived from three independently prepared solution A replicates, ensuring comparability with LC–UV analysis.

Specificity was evaluated by comparing the results of LC–UV quantification (absolute calibration method) with those of LC–MS/MS, which served as an orthogonal method. Absolute calibration results were expressed as the ratio of LC–UV to LC–MS/MS quantification and evaluated against the acceptance criteria described in AOAC Appendix K (AOAC International, 2023).

### 2.8 Examination of peak-identification procedures

The identity of the PMF3 peak in the KP solution was confirmed by analyzing uracil and the PMF3 standard solution under the same LC conditions used for quantification. A uracil solution (~10 μg/mL, dissolved in H_2_O/MeOH (50/50, v/v)) was used as the void marker (Rimmer et al., 2002). The retention time of PMF3 in the KP solution was compared with that of the PMF3 standard solution.

For the other PMFs, relative retention (*r*) was calculated from the retention time of PMF3 in the KP solution and the hold-up time determined using uracil, according to Eq. (4).

(4) $$r=\frac{t_{\mathrm{RB}}-t_{M}}{t_{\mathrm{RA}}-t_{M}}$$

where *r* is the relative retention of Peak B to Peak A, *t*_RA_ is the retention time of Peak A (PMF3 in the KP solution), *t*_RB_ is the retention time of the target PMF (Peak B in the KP solution), and *t*_M_ is the hold-up time determined using uracil. Based on a previous study (Iwasaki et al., 2023), PMF3 in the KP solution was first identified using the PMF3 standard. *r* was then defined using PMF3 in the KP solution, rather than the PMF3 standard, to improve reproducibility.

### 2.9 Quantification of PMFs by the RMS method

The PMF3 and KP solutions were analyzed individually by LC–UV at 265 nm. In the RMS method, the PMF concentrations in the KP solution were calculated using Eqs. (5), (6), based on a PMF3 calibration curve constructed as a proportional relationship passing through the origin after correction of the weighed amount for purity by ^1^H-qNMR.

(5) $$C_{1-6}=\frac{a_{1-6}}{\beta_{\mathrm{PMF}3}\times {\mathrm{rms}}_{1-6}}$$

(6) $${\mathrm{Ct}}_{1-6}=\frac{C_{1-6}\times M_{1-6}}{C_{\mathrm{PMF}3}}$$

where *C*_1–6_ represents the molar concentrations (mol/L) of PMF1–6 in the KP solution, *a*_1–6_ denotes the corresponding peak areas, *β*_PMF3_ is the regression coefficient of the PMF3 calibration curve, *rms*_1–6_ indicates the RMS value of PMF1–6 relative to PMF3, *Ct*_1–6_ refers to the content (%) of PMF1–6, *M*_1–6_ is the molecular weight (g/mol) of PMF1–6, and *C*_PMF3_ is the PMF3 concentration (mg/mL) of the standard solution. The total content was calculated as the sum of the individual PMF contents.

As described in Section 2.5, only a single concentration of PMF3 (~120 μmol/L) was prepared. In this case, the calculation is mathematically equivalent to a proportional conversion from a single-point calibration. However, the regression coefficient is reported here to maintain general applicability.

### 2.10 RMS method validation: accuracy and precision

The accuracy and precision of the RMS method were evaluated using recovery rates. For each PMF and for total PMFs, the recovery rate was calculated as the ratio of the RMS-based result to that obtained by the absolute calibration method under identical chromatographic conditions.

Accuracy was assessed by preparing KP solutions at three concentration levels (80%, 100%, and 120% of the target concentration) and analyzing each in triplicate using LC system A. Precision was evaluated over six days using a predefined staggered schedule involving two analysts (a and b), two LC systems (A and B), and two column lots (No. 2 and No. 3) (see Table S4). Two test solutions were prepared in parallel by each analyst and analyzed over three days, with LC systems and columns alternated according to the design. Relative standard deviations for repeatability (RSD_r_) and intermediate precision (RSD_int_) were calculated from one-way ANOVA results using recovery values.

Validation criteria for accuracy and precision were established with reference to AOAC Appendix K (AOAC International, 2023), and all tests were performed accordingly. System suitability was confirmed by replicate injections of PMF3, with %RSD calculated according to USP <621> (United States Pharmacopeia, 2024).

### 2.11 Uncertainty

The uncertainty of the RMS-based quantification was evaluated to ensure the reliability of the analytical results. Expanded uncertainty was estimated by considering contributions from RMS variability, intermediate precision, the purity of the PMF3 single-reference standard, molecular weights, and the measurement equipment used for sample preparation. A coverage factor of *k* = 2 was applied to obtain the expanded uncertainty.

## 3 Results

### 3.1 Determination of PMF solution concentrations by ^1^H-qNMR

**Fig. 1:**
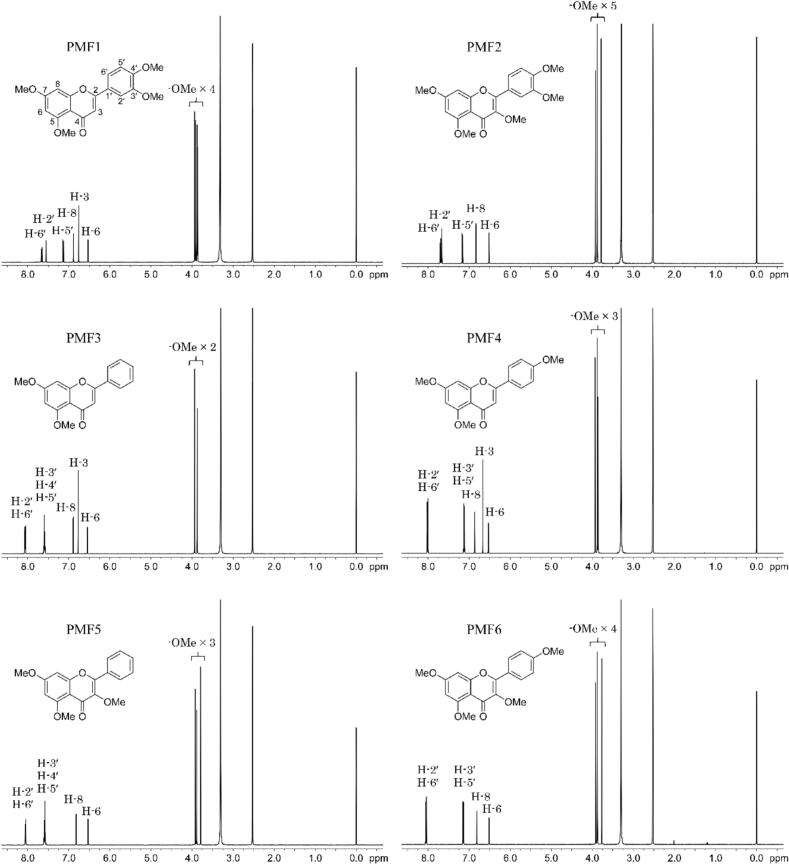
Chemical structures and ^1^H-qNMR spectra of PMFs acquired on a 500 MHz NMR system. DSS‑*d*_6_ was used as the internal standard and referenced at *δ* 0.00 ppm.

Each PMF sample was analyzed by ^1^H-qNMR using three independently prepared solution A samples for each PMF (Fig. S1). No obvious impurity signals were observed in the ^1^H-qNMR spectra of the six PMFs (Fig. 1). However, for PMF1 (7.14 ppm, H-5′) and PMF6 (3.77–3.92 ppm, -OMe × 4), potential overlap with interfering signals could not be excluded; therefore, these signals were omitted from quantification. The molar concentration of each solution A was then determined by averaging the values obtained from the remaining selected signals. Table 1 summarizes the quantitative signals, their assignments, and the resulting molar concentrations. These ^1^H-qNMR-based concentrations were used directly in subsequent LC–UV and LC–MS/MS analyses and for RMS determination. In addition, the purity of each PMF reagent was determined using the same ^1^H-qNMR approach. PMF3 exhibited a purity of 99.2 ± 0.3%, and the other PMFs showed comparably high purities, as summarized in Table S5.

**Table 1:** Molar concentrations of PMFs determined by ^1^H‑qNMR.

Compound	Position	Chemical Shift (*δ*, ppm)	Molar concentration of PMFs (μmol/L, %RSD)		
			1	2	3
PMF1	H-6′, H-2′	7.55, 7.66	7452	7736	7466
	H-5′	7.14	(7491)	(7796)	(7527)
	H-8, H-3	6.76, 6.89	7442	7753	7474
	H-6	6.53	7448	7737	7434
	-OMe × 4	3.86, 3.88, 3.91, 3.94	7449	7741	7470
		Average (selected signals)	7448 (0.1%)	7742 (0.1%)	7461 (0.2%)
PMF2	H-6′, H-2′	7.67, 7.70	7271	7444	7520
	H-5′	7.16	7276	7448	7513
	H-8	6.84	7271	7427	7500
	H-6	6.52	7278	7419	7490
	-OMe × 5	3.79–3.93	7272	7439	7501
		Average	7274 (0.0%)	7436 (0.2%)	7505 (0.2%)
PMF3	H-6′, H-2′	8.05–8.08	7523	7549	7638
	H-3′, H-4′, H-5′	7.57–7.62	7563	7562	7643
	H-8, H-3	6.77, 6.89	7545	7546	7638
	H-6	6.55	7526	7554	7622
	-OMe × 2	3.87, 3.94	7531	7547	7624
		Average	7537 (0.2%)	7552 (0.1%)	7633 (0.1%)
PMF4	H-6′, H-2′	8.01	7788	7696	7526
	H-3′, H-5′	7.12	7807	7708	7528
	H-8	6.87	7804	7723	7559
	H-3, H-6	6.53, 6.67	7822	7718	7550
	-OMe × 3	3.86, 3.88, 3.93	7806	7720	7538
		Average	7805 (0.2%)	7713 (0.1%)	7540 (0.2%)
PMF5	H-6′, H-2′	8.04–8.06	7506	7516	7626
	H-3′, H-4′, H-5′	7.55–7.62	7533	7522	7652
	H-8	6.82	7514	7522	7651
	H-6	6.53	7549	7506	7641
	-OMe × 3	3.79, 3.89, 3.92	7554	7539	7668
		Average	7531 (0.3%)	7521 (0.2%)	7648 (0.2%)
PMF6	H-6′, H-2′	8.05	7461	7520	7601
	H-3′, H-5′	7.14	7464	7521	7602
	H-8	6.81	7495	7511	7631
	H-6	6.51	7465	7534	7598
	-OMe × 4	3.77–3.92	(7516)	(7575)	(7674)
		Average (selected signals)	7472 (0.2%)	7522 (0.1%)	7608 (0.2%)

Molar concentrations were determined from three independent preparations of solution A; values are presented as averages (%RSD) across the assigned signals. For PMF1 (7.14 ppm, H‑5′) and PMF6 (3.77–3.92 ppm, –OMe × 4), signals potentially affected by overlap were excluded, and averages were calculated from the remaining selected signals. Chemical shifts were referenced to the DSS‑*d*_6_ signal at *δ* 0.00 ppm.

### 3.2 Chromatographic conditions

**Fig. 2:**
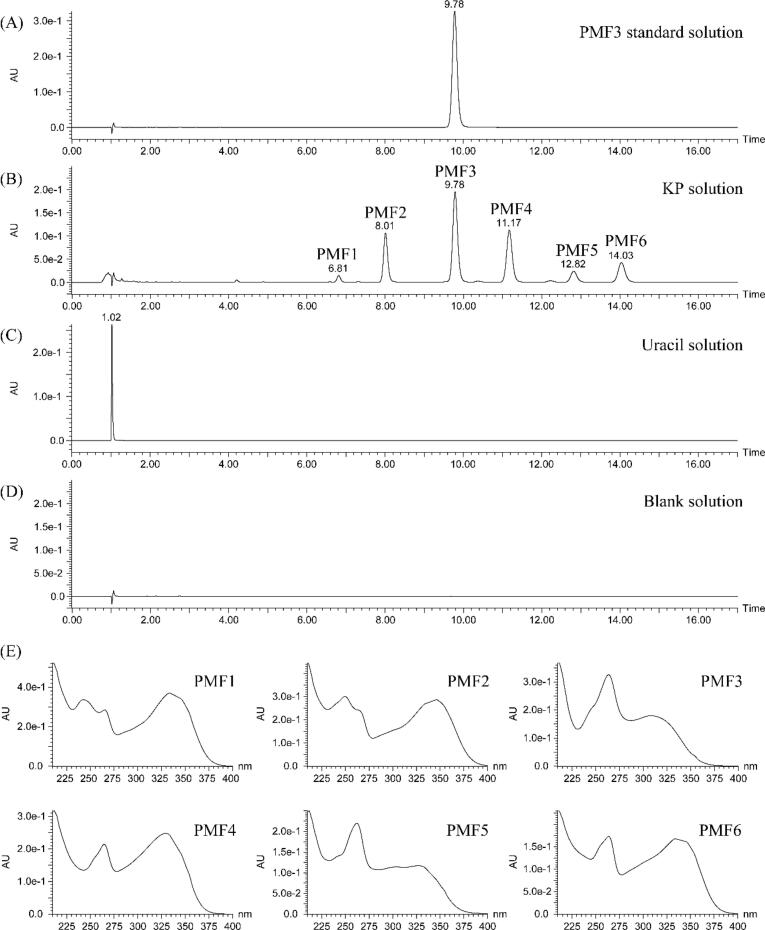
Representative chromatograms and UV spectra obtained under optimized LC conditions. (A–D) Chromatograms of the (A) PMF3 standard solution (Section 2.5), (B) KP solution (Section 2.5), (C) uracil solution (Section 2.8), and (D) blank solution (diluent, Section 2.4) obtained using LC system A. (E) UV spectra of PMF1–PMF6 obtained from solution C of each PMF using LC system B.

The chromatographic conditions were optimized to ensure high-resolution separation of the six PMFs in the KP extract. The Poroshell 120 EC-C18 (2.1 mm × 150 mm, 1.9 μm) column was selected based on preliminary evaluations using multiple reversed-phase columns and mobile phase systems. Although the H_2_O/MeOH system provided favorable separation, a ternary system (H₂O/MeCN/MeOH) was selected to ensure sufficient resolution for all six PMFs by the addition of a small amount of MeCN. The UV spectra of the six PMFs were compared, and all compounds exhibited stable absorbance at 265 nm, which was used for quantification. Representative chromatograms from LC system A are shown in Fig. 2. The reproducibility and consistency of the RMS values were verified across the three LC systems (A, B, and C) with different detection principles and designs.

Using the procedure described in Section 2.8, the relative retention (*r*) values for PMF1–6 were determined as follows: PMF1, 0.67; PMF2, 0.80; PMF4, 1.16; PMF5, 1.34; and PMF6, 1.49 (all relative to the PMF3 peak identified in the KP extract). Combining these values with the chromatogram shown in Fig. 2B enabled the reliable identification of all six PMFs in the KP solution, using PMF3 and uracil as references.

### 3.3 Selection of a single-reference standard

At the time of this study, all six target PMFs were available as reagents. PMF2, PMF5, and PMF6 are expensive and available from only a limited number of suppliers. In contrast, PMF1, PMF3, and PMF4 are commercially available at a lower cost. Comparison of UV sensitivity at 265 nm showed that PMF3 provided the highest detector response. PMF3 also exhibited negligible hygroscopicity, supporting its chemical stability and therefore its suitability as a reference standard. The high purity of PMF3 was confirmed using the ^1^H-qNMR approach (see Section 3.1). In addition, PMF3 is available from multiple manufacturers, supporting its practical use as a single-reference standard for RMS-based quantification. Accordingly, PMF3 was selected as the single-reference standard for subsequent RMS-based quantification based on its availability, stability, purity, and detector response.

### 3.4 Determination of RMS values and their applicable range

For each PMF, a calibration set comprising solutions C–H was prepared in triplicate and analyzed using LC systems A–C. Calibration plots were constructed with the molar concentration (determined by ^1^H-qNMR) on the x-axis and peak area on the y-axis. All calibration curves showed excellent linearity, with coefficients of determination (*R*^2^) of >0.999. The RMS values for each PMF relative to PMF3 were calculated as the average of nine measurements (three calibration sets × three LC systems), using the ratio of the regression coefficients obtained from the linear regression lines passing through the origin. The calibration ranges, regression coefficients, and RMS values are summarized in Table 2. The %RSD of the RMS values across the three instruments was ≤1.5%, and the three-measurement sets were comparable. The final RMS values adopted for PMF1–6 were as follows: PMF1, 0.655; PMF2, 0.651; PMF4, 0.748; PMF5, 0.850; and PMF6, 0.767 (PMF3 = 1.00).

**Table 2:** Regression analysis results for the determination of RMS relative to PMF3.

		System A					System B				System C			Average (%RSD)
		1	2	3	Ave. (%RSD)	1	2	3	Ave. (%RSD)	1	2	3	Ave. (%RSD)	
Column ID		No. 1	No. 1	No. 2		No. 1	No. 2	No. 1		No. 2	No. 1	No. 2		
PMF3	Range	1.93–121	1.93–121	1.95–122		1.93–121	1.93–121	1.95–122		1.93–121	1.93–121	1.95–122		1.95–121 μmol/L
	*β*	405.732	397.566	408.188		392.446	393.812	394.578		23.1504	22.7300	22.5321		
	*R*^2^	0.99999	0.99999	0.99999		0.99998	0.99998	0.99998		0.99998	0.99999	0.99999		
PMF1	Range	1.91–119	1.98–124	1.91–119		1.91–119	1.98–124	1.91–119		1.91–119	1.98–124	1.91–119		1.98–119 μmol/L
	*β*	259.551	257.226	265.199		255.882	260.309	263.818		15.0418	14.9767	15.0174		
	*R*^2^	0.99999	1.00000	0.99996		0.99998	0.99999	0.99999		0.99994	0.99999	0.99999		
	*rms*	0.6397	0.6470	0.6497	0.645 (0.8%)	0.6520	0.6610	0.6686	0.661 (1.3%)	0.6497	0.6589	0.6665	0.658 (1.3%)	0.655 (1.5%)
PMF2	Range	1.86–116	1.90–119	1.92–120		1.86–116	1.90–119	1.92–120		1.86–116	1.90–119	1.92–120		1.92–116 μmol/L
	*β*	262.279	254.969	265.847		255.021	254.322	258.232		15.1149	14.8775	14.9660		
	*R*^2^	0.99993	0.99999	0.99999		0.99999	0.99999	1.00000		0.99995	0.99986	0.99997		
	*rms*	0.6464	0.6413	0.6513	0.646 (0.8%)	0.6498	0.6458	0.6545	0.650 (0.7%)	0.6529	0.6545	0.6642	0.657 (0.9%)	0.651 (1.0%)
PMF4	Range	2.00–125	1.97–123	1.93–121		2.00–125	1.97–123	1.93–121		2.00–125	1.97–123	1.93–121		2.00–121 μmol/L
	*β*	297.120	295.576	304.739		292.222	298.947	300.780		17.0537	17.0060	17.0018		
	*R*^2^	0.99998	0.99999	0.99997		0.99998	0.99997	0.99995		0.99998	0.99996	0.99999		
	*rms*	0.7323	0.7435	0.7466	0.741 (1.0%)	0.7446	0.7591	0.7623	0.755 (1.2%)	0.7366	0.7482	0.7546	0.746 (1.2%)	0.748 (1.3%)
PMF5	Range	1.93–121	1.93–120	1.96–122		1.93–121	1.93–120	1.96–122		1.93–121	1.93–120	1.96–122		1.96–120 μmol/L
	*β*	342.752	343.244	349.561		328.472	334.680	331.688		19.6034	19.4937	19.2977		
	*R*^2^	0.99999	0.99998	1.00000		0.99998	0.99999	0.99999		0.99999	0.99993	0.99997		
	*rms*	0.8448	0.8634	0.8564	0.855 (1.1%)	0.8370	0.8498	0.8406	0.842 (0.8%)	0.8468	0.8576	0.8565	0.854 (0.7%)	0.850 (1.0%)
PMF6	Range	1.91–120	1.93–120	1.95–122		1.91–120	1.93–120	1.95–122		1.91–120	1.93–120	1.95–122		1.95–120 μmol/L
	*β*	306.469	306.948	315.019		299.074	305.654	304.968		17.5954	17.3449	17.3954		
	*R*^2^	0.99999	0.99999	0.99999		0.99999	0.99999	0.99998		0.99999	0.99999	0.99999		
	*rms*	0.7553	0.7721	0.7718	0.766 (1.3%)	0.7621	0.7761	0.7729	0.770 (1.0%)	0.7600	0.7631	0.7720	0.765 (0.8%)	0.767 (0.9%)

Each result corresponds to an independently prepared solution from which a six-level calibration set (solutions C—H) was prepared. Range: concentration range of solutions used for RMS determination; *β*: regression coefficient; *R*^*2*^: coefficient of determination; *rms*: RMS value of each PMF relative to PMF3 (by definition, PMF3 = 1.00). The column IDs correspond to those listed in Supplementary Table S2.

All data from solution H, which represented the lowest concentration in the calibration series for each PMF, showed S/N ratios of ≥10. Therefore, the RMS values determined in this study are applicable at concentrations of 2.00 μmol/L and above, consistent with the measured concentrations of solution H for each PMF. Although this method is not intended for use near the detection limit, the limit of detection (LOD) was estimated for convenience as 0.3 × the lowest concentration. Accordingly, the LOQ and LOD were concluded to be 2.00 μmol/L and 0.600 μmol/L, respectively.

### 3.5 Specificity evaluation of PMFs

LC–UV quantification was conducted concurrently with RMS determination using the same calibration curve, as described in Section 2.6. These LC–UV results served two purposes: comparison with LC–MS/MS to evaluate specificity and comparison with the RMS method to assess accuracy and precision.

LC–MS/MS quantification was performed separately from the LC–UV measurements, as described in Section 2.7. Analyses were conducted in the MRM mode using transitions optimized for each PMF. The KP solution was diluted 20-fold and analyzed in triplicate by LC–MS/MS using a reduced injection volume (0.1 μL). In combination with chromatographic separation, these transitions enabled selective detection, including compounds with identical molecular weights. The extracted ion chromatograms (EICs) for each MRM transition, obtained from the 20-fold diluted KP solution, are shown in Fig. S2. PMF1 and PMF6 were not detected exclusively under their corresponding MRM transitions; however, chromatographic resolution was sufficient to prevent interference during quantification. By contrast, PMF4 and PMF5 were detected with near-complete exclusivity under their corresponding transitions.

By combining these optimized transitions with high-resolution LC conditions, the method achieved high specificity for compound detection. For quantification, a linear calibration curve with 1/x weighting was constructed for each PMF using the corresponding EIC, without constraining the intercept to the origin. This approach was selected to reduce signal saturation and variability at higher concentrations, thereby improving accuracy across the entire calibration range. The LC–MS/MS calibration curves exhibited excellent linearity (*R*^2^>0.997) throughout the study.

The ratios of LC–UV to LC–MS/MS results for each PMF and for total PMFs were used to evaluate specificity (Table 3). The differences between the LC–UV and LC–MS/MS results were well within the acceptance criteria with reference to Appendix K of the AOAC Official Methods of Analysis (AOAC International, 2023).

**Table 3:** PMF contents of the KP solution determined by LC–UV (absolute calibration method) and LC–MS/MS, with the ratio between them (LC–UV/LC–MS/MS, %).

Compound	LC–UV		LC–MS/MS		Ratio (%)
	Average	(%RSD)	Average	(%RSD)	
PMF1	0.273%	(0.4%)	0.274%	(3.4%)	99.5%
PMF2	2.44%	(0.8%)	2.44%	(4.0%)	99.8%
PMF3	2.59%	(0.9%)	2.61%	(3.3%)	98.9%
PMF4	2.55%	(0.4%)	2.53%	(4.0%)	100.6%
PMF5	0.541%	(0.9%)	0.552%	(3.6%)	98.0%
PMF6	1.28%	(0.6%)	1.30%	(3.3%)	98.6%
Total PMFs	9.67%	(0.5%)	9.71%	(3.3%)	99.5%

The quantification procedures are described in 2.6, 2.7.

### 3.6 RMS method validation: accuracy and precision

System repeatability was confirmed prior to accuracy evaluation; the %RSD values were below 1%, meeting the acceptance criteria. The accuracy of the RMS method was assessed by determining recovery at three concentration levels (80%, 100%, and 120%) using the KP solutions and comparing the results with those obtained using the absolute calibration method (Table 4). Recoveries ranged from 96.3% to 100.0% and were consistent across all concentration levels. All results met the acceptance criteria with reference to the numerical ranges specified in AOAC Appendix K, supporting the validity of the RMS method relative to the absolute calibration approach.

**Table 4:** Validation of the accuracy and precision of the RMS method.

	Accuracy					Precision		Uncertainty
Compound	Level	Average	(%RSD)	Total Ave. (%RSD)	Min / Max	RSD_r_	RSD_int_	U (*k* = 2)
PMF1	80% (32 mg)	97.7%	(0.6%)	97.4%	96.3%			
	100% (40 mg)	97.1%	(1.0%)	(0.8%)	/ 98.5%	0.33%	1.08%	3.81%
	120% (48 mg)	97.6%	(0.8%)					
PMF2	80% (32 mg)	98.5%	(0.6%)	98.1%	96.7%			
	100% (40 mg)	97.5%	(0.8%)	(0.8%)	/ 99.2%	0.35%	0.35%	2.31%
	120% (48 mg)	98.2%	(1.0%)					
PMF3	80% (32 mg)	99.5%	(0.4%)	99.1%	98.1%			
	100% (40 mg)	98.7%	(0.8%)	(0.7%)	/ 100.0%	0.36%	0.98%	2.16%
	120% (48 mg)	99.2%	(0.7%)					
PMF4	80% (32 mg)	97.9%	(0.5%)	97.6%	96.5%			
	100% (40 mg)	97.1%	(0.8%)	(0.7%)	/ 98.4%	0.35%	0.63%	3.38%
	120% (48 mg)	97.6%	(0.7%)					
PMF5	80% (32 mg)	99.3%	(0.5%)	98.8%	97.6%			
	100% (40 mg)	98.3%	(0.9%)	(0.7%)	/ 99.8%	0.29%	0.84%	2.53%
	120% (48 mg)	98.8%	(0.8%)					
PMF6	80% (32 mg)	98.7%	(0.5%)	98.1%	96.9%			
	100% (40 mg)	97.7%	(0.8%)	(0.8%)	/ 99.2%	0.34%	0.42%	2.63%
	120% (48 mg)	97.9%	(0.9%)					
Total PMFs	80% (32 mg)	98.7%	(0.5%)	98.3%	97.1%			
	100% (40 mg)	97.9%	(0.9%)	(0.7%)	/ 99.3%	0.34%	0.38%	1.27%
	120% (48 mg)	98.3%	(0.8%)					

Accuracy was assessed as recovery of the RMS-based results relative to those obtained by the absolute calibration method using LC–UV, evaluated in triplicate (*n* = 3) at each concentration level. Precision was evaluated using two independent test solutions (*n* = 2) prepared by each analyst and analyzed over three days per analyst (six days in total), and assessed by calculating recovery rates relative to the LC–UV results using one‑way ANOVA.

Total Ave.: Average calculated from all three levels; Min/Max: Minimum and maximum values calculated from all three levels; RSD_r_: Relative standard deviation of repeatability; RSD_int_: Intermediate precision; U: Expanded uncertainty; *k*: Coverage factor.

Precision was evaluated using a predefined experimental design involving multiple instruments, analysts, and column lots. System repeatability met the acceptance criteria for all measurements, with %RSD values below 1%. As shown in Table 4, both RSD_r_ and RSD_int_ met the criteria with reference to the numerical ranges specified in AOAC Appendix K, confirming that the RMS method provides sufficient precision for practical application.

For all PMFs, the expanded uncertainty (*k* = 2) was ≤3.81%, supporting the reliability of the RMS-based quantification (Table 4). The contributions from the purity of the PMF3 single-reference standard, molecular weights, and mass measurements to the expanded uncertainty were limited.

## 4 Discussion

In this study, RMS-based quantification was applied to six PMFs in the KP extract, enabling accurate measurement using PMF3 as a single-reference standard. Unlike previous reports that relied on individually isolated (Ninomiya et al., 2016) or commercially available standards (Joothamongkhon et al., 2022), the present method enables precise quantification of all six PMFs using only one reference standard. This approach simplifies standard management and reduces costs, addressing a key limitation in both research and industrial settings where multiple reference standards are not readily available.

To clarify the positioning of the RMS-based approach relative to the conventional absolute calibration method, their key characteristics are summarized in Table 5. RMS-based quantification requires fixed chromatographic conditions, including defined mobile-phase composition and detection wavelength, which can limit flexibility across diverse sample types (Iwasaki et al., 2023). Accordingly, the RMS values obtained in this study are applicable only under the fixed detection wavelength of 265 nm and the mobile phase conditions employed herein. Nevertheless, because the KP extract is incorporated into various food formats, it is essential to quantify PMFs in complex matrices. To achieve high resolution, a sub-2-μm superficially porous particle column [Poroshell 120 EC-C18 (2.1 mm × 150 mm, 1.9 μm)] was adopted, and the mobile-phase conditions were optimized with reference to a previously reported approach that achieved excellent separation using 1.7 μm superficially porous particles (Fekete & Guillarme, 2013).

**Table 5:** Comparison of the RMS and conventional absolute calibration methods.

Category	RMS method (this study)	Conventional absolute calibration method
Standards	Single‑reference standard (PMF3) with purity assurance by ^1^H‑qNMR	All individual standards (PMF1–PMF6) with purity assurance by ^1^H‑qNMR
Method applicability	Single‑reference standard selected based on stability and availability	Depends on the availability of all individual standards
Handling	Preparation and analysis based on a single-reference standard	Preparation and analysis involving multiple individual standard solutions and/or mixed standard solutions
Chromatographic conditions	RMS factor applied under fixed mobile‑phase composition and detection wavelength	Mobile phase composition and detection wavelength can be selected according to the analytical purpose
Gradient elution	Should be applied cautiously, considering inter‑instrument variability and analytical purpose	Applicable
Peak identification	Routine identification based on relative retention, with optional confirmation by UV spectra or identification analytes	Identification based on comparison with individual standards
Uncertainty	Expanded uncertainty includes the uncertainty of individual RMS values for each PMF	Expanded uncertainty derived from calibration and preparation steps of six individual PMF standards

Previous studies have shown that RMS-based quantification is sensitive to detector settings and analyte spectral properties; differences in detection wavelength and spectral characteristics can affect reproducibility, particularly in HPLC/PDA systems (Nishizaki et al., 2020b). Classical studies established that detector bandwidth influences absorbance linearity in UV and PDA detectors (Vial & Jardy, 1999), and that the spectral resolution of PDA detectors depends on instrument design, including the number of diodes (Riordon, 2000). These principles remain applicable to modern instruments and highlight the importance of selecting appropriate detection wavelengths (e.g., absorption maxima) to minimize instrument-to-instrument variability in RMS-based measurements. Consistent with this concept, several RMS-related studies have evaluated approaches that use the absorption maxima of both the reference standards and analytes with different UV spectral profiles (Nishizaki et al., 2018; Ohtsuki et al., 2020, 2024). In the present study, which aimed to simultaneously quantify six PMFs in the KP extract, the use of a single detection wavelength was more practical than using individual absorption maxima because one wavelength can be applied consistently with both PDA and UV detectors.

To identify an appropriate wavelength, the UV spectra of the six PMFs were visually compared (Fig. 2E). Based on the stability of absorbance around local maxima, local minima, and plateau-like regions, 265 nm was selected. Nevertheless, slight spectral differences near 265 nm could still introduce instrument-dependent variability, and this possibility cannot be fully excluded. To address this concern, RMS values were determined using three LC instruments from different vendors with different detection principles, as well as two column lots. The resulting RMS values showed low %RSD, confirming high reproducibility and practical applicability. To accommodate multiple detector types, LC system C was used for RMS measurements. However, because LC system C operated near its pressure limit under the optimized analytical conditions and was not suitable for routine analysis, method validation was performed using LC systems A and B. Based on these results, the RMS values can be applied without routine re-calibration under equivalent chromatographic conditions. However, as a precaution, verification of comparable performance is recommended when introducing new instruments or during initial implementation in a different laboratory.

The JAS guidelines outline general requirements for reference standards used in RMS-based quantification (Ministry of Agriculture, Forestry and Fisheries, 2025); however, they do not specify which compounds should be used in practice. Therefore, selecting an appropriate reference standard still requires compound-specific evaluation. The guidelines state that reference standards must have accurately determined purity—typically established by techniques such as qNMR—must be stable and readily obtainable, and must be chromatographically separable from the target analytes during RMS measurement. In particular, metrological traceability of the purity value is desirable. In this study, PMF3 was selected as the reference standard. It showed no stability issues during the experiments and is available from multiple vendors at a reasonable cost. Furthermore, the PMF3 reagent used in this study exhibited high purity (99.2%), as determined by ^1^H-qNMR. Because a CRM of DSS‑*d*_6_ was used for the ^1^H-qNMR measurement, traceability to the International System of Units (SI) was ensured. If PMF3 becomes available as a CRM in the future, its metrological traceability will be further strengthened.

In practice, peak identification can be challenging because the RMS method does not require all standards to be analyzed in every run. Using all six PMF standards each time would undermine the practical advantages of the RMS approach. Therefore, following previous reports (Iwasaki et al., 2023; Mizumoto et al., 2019), we implemented a peak-identification option based on relative retention. For UV detectors, peaks can be identified either by analyzing a KP extract validated to contain all six PMFs or by using PMF standards only during the initial setup. Once retention times have been established, routine peak identification can be performed reliably using relative retention without including all standards in every run. For PDA systems, UV spectral matching can be combined with relative retention to further support peak identification.

Specificity was confirmed by the agreement between LC–UV and LC–MS/MS results, obtained using two orthogonal detection principles. This supports that co-eluting compounds and matrix components in the KP extract did not have a practical impact on quantification under the applied reversed-phase UHPLC conditions, consistent with previous reports (Joothamongkhon et al., 2022; Ninomiya et al., 2016). Accuracy was demonstrated by recoveries of 96–100% across three concentration levels, while both repeatability and intermediate precision met acceptance criteria established with reference to the AOAC guidelines. The expanded uncertainty (*k* = 2) was ≤3.81% for all PMFs. Collectively, these results confirm the practical applicability of the newly designed RMS-based method for routine simultaneous quantification of PMFs in the KP extract, highlighting the need for experimental validation beyond theoretical optimization.

## 5 Conclusion

The validated RMS-based LC–UV method developed in this study enables reliable quantification of six PMFs in the KP extract, supporting applications ranging from fundamental research to routine quality control. The method established here is primarily intended for KP extracts prepared from plant materials and for KP extracts used as food ingredients. In Japan, the FFC system allows functional claims not only for dietary supplements but also for general foods, implying that the KP extract may be incorporated into diverse and complex food matrices. Although the present study focused on a single KP extract, the optimized UHPLC conditions established here are expected to provide a basis for quantitative analysis under such applications, provided that matrix-specific verification is performed. Practical considerations, including the use of relative retention for peak identification, were also addressed to facilitate immediate implementation in routine workflows. By simplifying standard management and mitigating challenges associated with the limited availability and high cost of high-purity reference standards, this method offers a practical solution for regulatory frameworks such as the FFC system.

Although validation was conducted with reference to international guidelines, further studies are warranted to assess interlaboratory precision and applicability across a broader range of matrices. In this context, integrating LC–MS/MS—used here for specificity assessment—into RMS-based quantification represents a promising direction for future development. This approach could extend the RMS methodology to complex matrices and trace-level analyses. However, because LC–MS/MS generally exhibits lower reproducibility than LC–UV, additional work is required to address this limitation before practical implementation. Collectively, these results provide a strong foundation for extending RMS-based quantification to regulatory and quality control applications for KP-derived products in both domestic and international markets.

## CRediT authorship contribution statement

**Daigo Iwasaki:** Writing – original draft, Visualization, Validation, Investigation, Formal analysis, Conceptualization. **Hiroaki Kawamoto:** Writing – review & editing, Validation, Resources. **Toshiyuki Murakami:** Writing – review & editing, Project administration, Investigation, Conceptualization. **Takashi Ohtsuki:** Writing – review & editing, Methodology. **Hiroshi Matsufuji:** Writing – review & editing, Supervision, Methodology.

## Declaration of generative AI and AI-assisted technologies in the writing process

During the preparation of this manuscript, the authors used Microsoft Copilot for Microsoft 365 to improve its language and readability. After using this tool, the authors reviewed and edited the content as required and take full responsibility for the content of the published article.

## Funding

This research was funded by Maruzen Pharmaceuticals Co., Ltd.

## Declaration of competing interest

The authors declare the following financial interests/personal relationships which may be considered as potential competing interests: Daigo Iwasaki reports financial support and travel were provided by Maruzen Pharmaceuticals Co., Ltd. Daigo Iwasaki reports a relationship with Maruzen Pharmaceuticals Co., Ltd. that includes: employment and travel reimbursement. Hiroaki Kawamoto reports a relationship with Maruzen Pharmaceuticals Co., Ltd. that includes: employment. Toshiyuki Murakami reports a relationship with Maruzen Pharmaceuticals Co., Ltd. that includes: employment. Daigo Iwasaki has patent pending to Maruzen Pharmaceuticals Co., Ltd. If there are other authors, they declare that they have no known competing financial interests or personal relationships that could have appeared to influence the work reported in this paper.

## Data Availability

Data will be made available on request.

## Glossary

ANOVA: analysis of variance; CRM: certified reference material; FA: formic acid; FFC: foods with function claims; KP: *Kaempferia parviflora*; LC–MS/MS: liquid chromatography–tandem mass spectrometry; MeCN: acetonitrile; MeOH: methanol; PDA: photodiode array; PMF: polymethoxyflavone; qNMR: quantitative nuclear magnetic resonance; RMS: relative molar sensitivity; UHPLC: ultrahigh-performance liquid chromatography.
