# Supplementary material for: Newly designed liquid chromatographic method using relative molar sensitivity based on 1H-qNMR for quantifying polymethoxyflavones from Kaempferia parviflora
Source: Food Chem X. 2026 Jun 18;37:104115. doi: 10.1016/j.fochx.2026.104115 (PMC13320506; doi:10.1016/j.fochx.2026.104115)
Supplement: Supplementary file 1 — Supplementary material [file mmc1.docx]

**SUPPLEMENTARY MATERIAL**

**Newly Designed Liquid Chromatographic Method Using Relative Molar Sensitivity Based on ^1^H‑qNMR for Quantifying Polymethoxyflavones from *Kaempferia parviflora***

Daigo Iwasaki^a,b,^*, Hiroaki Kawamoto^b^, Toshiyuki Murakami^b^, Takashi Ohtsuki^a^, Hiroshi Matsufuji^a^

^a^Department of Food Science and Technology, Graduate School of Bioresource Sciences, Nihon University, 1866, Kameino, Fujisawa-City, Kanagawa 252-0880, Japan

^b^Research Center, Maruzen Pharmaceuticals, Co., Ltd., 1089-8, Sagata, Shinnichi-Cho, Fukuyama-City, Hiroshima 729-3102, Japan

*Corresponding author.

E-mail address: [d-iwasaki@maruzenpcy.co.jp](mailto:d-iwasaki@maruzenpcy.co.jp) (D. Iwasaki)

**Table S1. Specifications of LC systems used in this study**

| Item / Parameter | LC System A | LC System B | LC System C |
| --- | --- | --- | --- |
| Instrument name | ACQUITY UPLC  H‑Class PLUS | ACQUITY UPLC  H‑Class PLUS | 1260 Infinity II Prime LC |
| Manufacturer | Waters | Waters | Agilent Technologies |
| Maximum pressure | 15,000 psi (~100 MPa) | 15,000 psi (~100 MPa) | 800 bar (80 MPa) |
| Pump | Quaternary Solvent Manager (QSM) | Binary Solvent Manager (BSM) | Quaternary Pump (G7104C Flexible Pump) |
| Autosampler | SM‑FTN‑H  (flow-through needle) | SM‑FTN‑H  (flow-through needle) | G7129C |
| Column oven | CH‑A | CH‑A | G7116A |
| Detector | Tunable UV Detector (TUV) | Photodiode Array Detector (PDAeλ) | Photodiode Array Detector (G7117C DAD) |
| Software | MassLynx (v4.2) | MassLynx (v4.2) | OpenLab CDS ChemStation Edition (vC.01.10) |
| Detector type | UV | PDA | PDA |
| Light source | D2 | D2 & W | D2 |
| Optical path length | 10 mm | 10 mm | 10 mm |
| Data sampling | 20 points/s (50 ms) | 20 points/s (50 ms) | 2.5 Hz (400 ms) |
| Wavelength (2D) | 265 nm | 265 nm | 265 nm |
| Bandwidth (2D) | <5 nm | 1.2 nm | 4 nm |
| Wavelength range (3D) | Single wavelength | 210–400 nm | 190–400 nm |
| Spectral resolution (3D) | Not applicable | 1.2 nm | 1 nm |
| Detection unit | 2 silicon photodiodes | 512 photodiode array | 1024 photodiode array |

**Table S2. Column lots and their usage in method development and validation.**

| Column ID | Lot No. | Usage |
| --- | --- | --- |
| No. 1 | B23458 | RMS determination, LC–UV analysis, LC–MS/MS analysis |
| No. 2 | B24184 | RMS, LC–UV, LC–MS/MS, accuracy, precision |
| No. 3 | B25031 | precision |

Note: The column IDs correspond to those in the RMS‑determination tables to ensure the traceability of the results.

**Table S3. LC–MS/MS MRM transitions and MS parameters**

| Compound | Precursor ion (*m*/*z*) | Product ion (*m*/*z*) | Collision energy (V) |
| --- | --- | --- | --- |
| PMF1 | 343.3 | 167.3 | 36 |
| PMF2 | 373.3 | 312.3 | 28 |
| PMF3 | 283.3 | 225.3 | 32 |
| PMF4 | 313.3 | 212.3 | 50 |
| PMF5 | 313.3 | 252.4 | 24 |
| PMF6 | 343.3 | 282.4 | 26 |

Conditions: capillary voltage, 3.0 kV; source temperature, 150 °C; cone voltage, 60 V; dwell time for all transitions, 0.022 s.

**Table S4. Experimental design for precision testing using a** **predefined staggered schedule.**

| Measurement No. | Analyst | LC system | Column ID |
| --- | --- | --- | --- |
| 1 | a | A | No. 3 |
| 2 | a | B | No. 3 |
| 3 | a | A | No. 2 |
| 4 | b | B | No. 2 |
| 5 | b | A | No. 2 |
| 6 | b | B | No. 3 |

Note: The table lists the measurement sequence, analyst (a and b), LC system (A and B), and column ID (Nos. 2 and 3) used for each analysis. For precision evaluation, two independent test solutions (n = 2) were prepared by each analyst on the first day and analyzed over three days per analyst (six days in total). The combinations were alternated according to a predefined schedule, and the column IDs correspond to those listed in Supplementary Table S2. Analyses were performed on different days in a shut‑down/restart sequence to reflect between‑day variation

**Table S5. Absolute purity of each PMF measured by ^1^H‑qNMR.**

| Compound | Position | Chemical Shift (*δ*, ppm) |  | Absolute Purity (%, Purity ± SD) | |  |
| --- | --- | --- | --- | --- | --- | --- |
|  |  |  |  |  | Average |  |
| PMF1 | H‑6′, H‑2′ | 7.55, 7.66 |  | 99.09 ± 0.06 | 99.1 ± 0.2 |  |
|  | H‑5′ | 7.14 |  | 99.79 ± 0.14 |  |  |
|  | H‑8, H‑3 | 6.76, 6.89 |  | 99.16 ± 0.14 |  |  |
|  | H‑6 | 6.53 |  | 98.94 ± 0.21 |  |  |
|  | ‑OMe × 4 | 3.86, 3.88, 3.91, 3.94 |  | 99.11 ± 0.04 |  |  |
| PMF2 | H‑6′, H‑2′ | 7.67, 7.70 |  | 95.93 ± 0.30 | 95.8 ± 0.3 |  |
|  | H‑5′ | 7.16 |  | 95.94 ± 0.21 |  |  |
|  | H‑8 | 6.84 |  | 95.77 ± 0.22 |  |  |
|  | H‑6 | 6.52 |  | 95.73 ± 0.20 |  |  |
|  | ‑OMe × 5 | 3.79–3.93 |  | 95.83 ± 0.17 |  |  |
| PMF3 | H‑6′, H‑2′ | 8.05–8.08 |  | 99.18 ± 0.44 | 99.2 ± 0.3 |  |
|  | H‑3′, H‑4′, H‑5′ | 7.57–7.62 |  | 99.43 ± 0.21 |  |  |
|  | H‑8, H‑3 | 6.77, 6.89 |  | 99.27 ± 0.28 |  |  |
|  | H‑6 | 6.55 |  | 99.15 ± 0.35 |  |  |
|  | ‑OMe × 2 | 3.87, 3.94 |  | 99.14 ± 0.30 |  |  |
| PMF4 | H‑6′, H‑2′ | 8.01 |  | 99.24 ± 0.18 | 99.4 ± 0.3 |  |
|  | H‑3′, H‑5′ | 7.12 |  | 99.38 ± 0.09 |  |  |
|  | H‑8 | 6.87 |  | 99.57 ± 0.29 |  |  |
|  | H‑3, H‑6 | 6.53, 6.67 |  | 99.58 ± 0.17 |  |  |
|  | ‑OMe × 3 | 3.86, 3.88, 3.93 |  | 99.47 ± 0.13 |  |  |
| PMF5 | H‑6′, H‑2′ | 8.04–8.06 |  | 99.32 ± 0.10 | 99.5 ± 0.3 |  |
|  | H‑3′, H‑4′, H‑5′ | 7.55–7.62 |  | 99.57 ± 0.07 |  |  |
|  | H‑8 | 6.82 |  | 99.48 ± 0.15 |  |  |
|  | H‑6 | 6.53 |  | 99.52 ± 0.25 |  |  |
|  | ‑OMe × 3 | 3.79, 3.89, 3.92 |  | 99.81 ± 0.06 |  |  |
| PMF6 | H‑6′, H‑2′ | 8.05 |  | 98.49 ± 0.04 | 98.6 ± 0.2 |  |
|  | H‑3′, H‑5′ | 7.14 |  | 98.51 ± 0.05 |  |  |
|  | H‑8 | 6.81 |  | 98.73 ± 0.33 |  |  |
|  | H‑6 | 6.51 |  | 98.56 ± 0.12 |  |  |
|  | ‑OMe × 4 | 3.77, 3.88, 3.88, 3.92 |  | (99.29 ± 0.11) |  |  |

Note: Chemical shifts are referenced to the DSS‑*d*_6_ signal as *δ* 0.00 ppm. For each signal, Purity ± SD was obtained from triplicate measurements (n = 3). For PMF1 (7.14 ppm, H‑5′) and PMF6 (3.77–3.92 ppm, –OMe × 4), signals potentially affected by overlap were excluded, and averages were calculated from the remaining selected signals. The SD values presented in the Average column were calculated from all individual purity measurements (n = 3 per signal) pooled across the assigned signals.


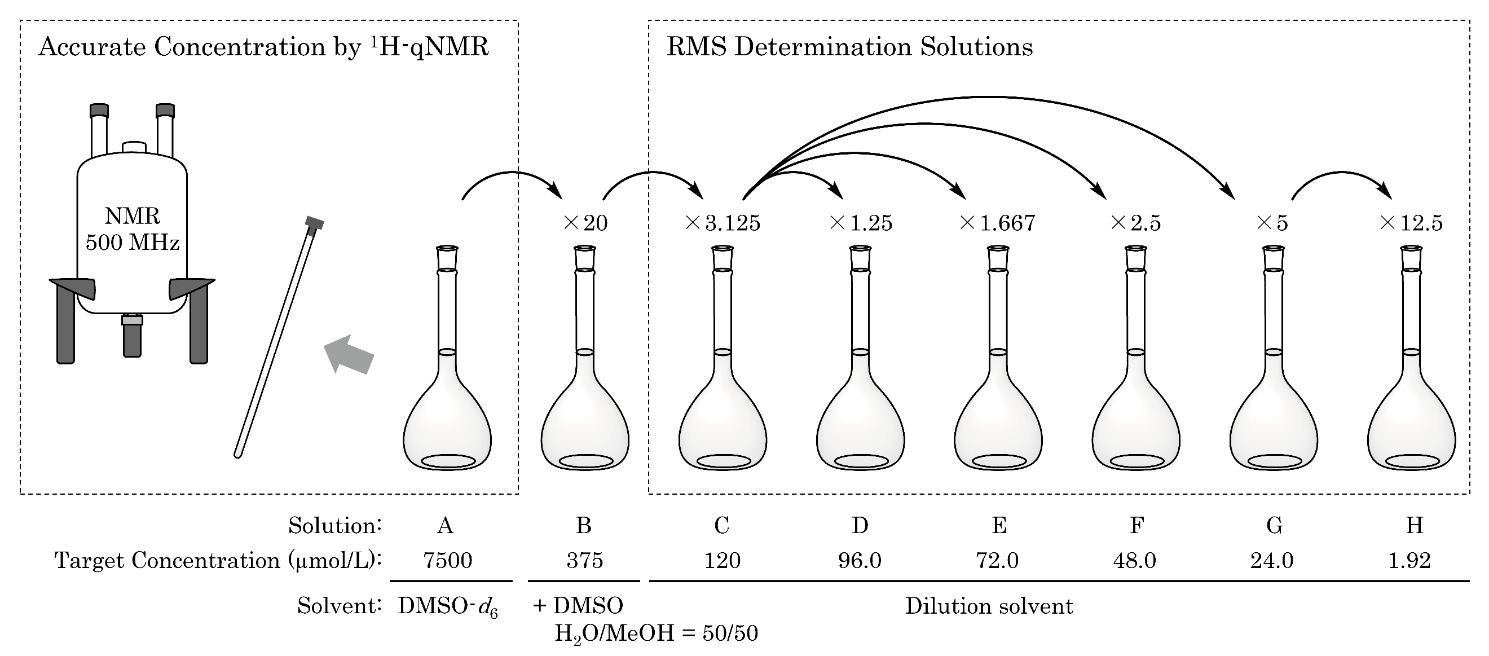


**Fig. S1. Schematic of the preparation of RMS determination solutions by serial dilution of stock solutions, with actual concentrations determined by ^1^H‑qNMR.** Solution A was prepared at the concentration determined through ^1^H‑qNMR (500 MHz, DMSO‑*d*_6_). Solution B was obtained by mixing 1 mL of solution A with 2 mL of DMSO, followed by the gradual addition of H_2_O/MeOH (50/50, v/v) upon stirring and adjustment of the total volume to 20 mL. For subsequent dilutions (solutions C–H), a diluent was prepared by combining 15 mL of DMSO with H_2_O/MeOH (50/50, v/v) and adjusting the volume to 100 mL. Because DMSO‑*d*_6_ is not practical for use in LC analysis, DMSO was used as a substitute for all subsequent dilutions. Solutions C–H were used as a six‑level calibration set for RMS determination.


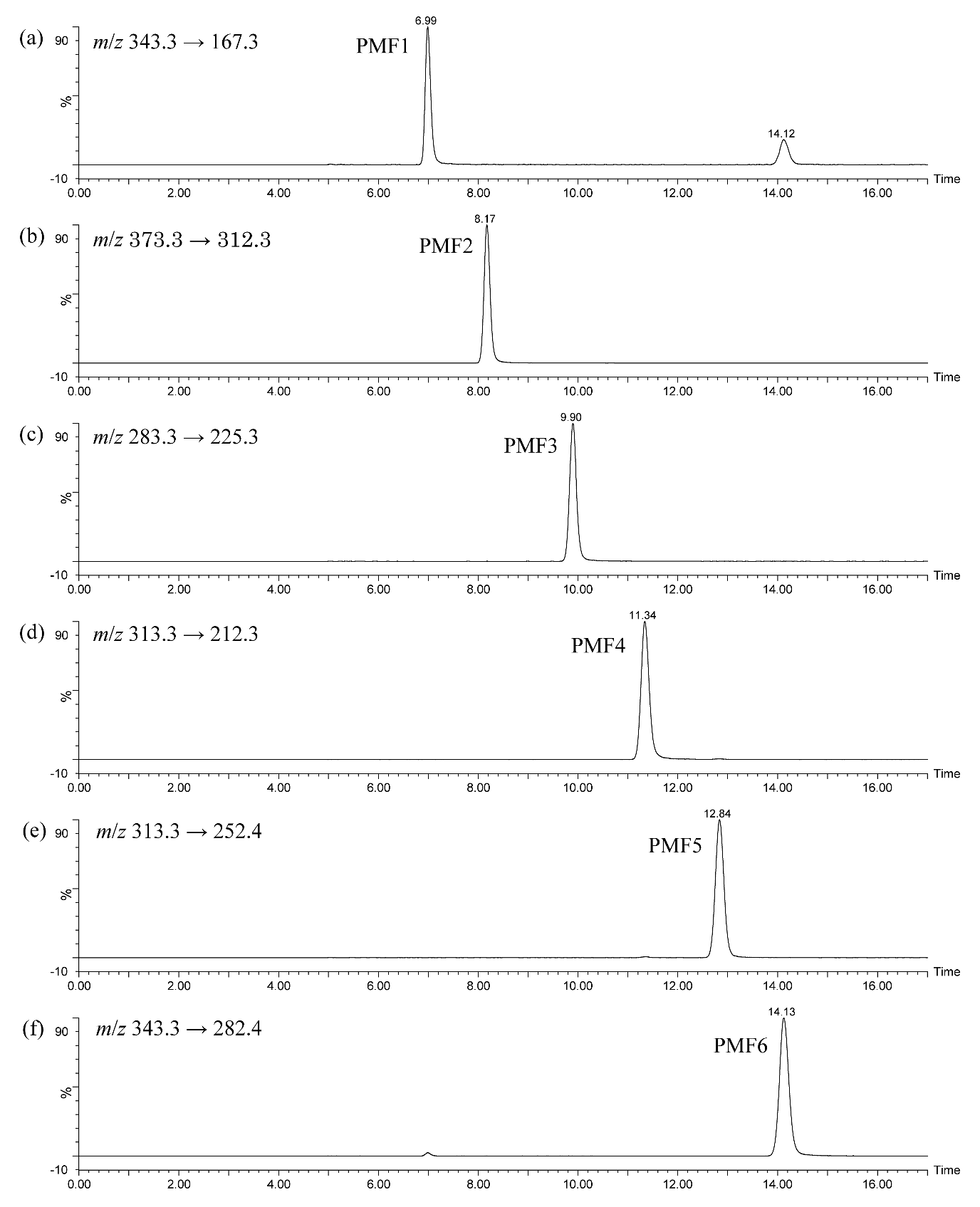


**Fig. S2. Extracted ion chromatograms for the MRM transitions of PMF1–PMF6.** A KP solution diluted 20‑fold was used as the test solution. All chromatograms are displayed at full scale.
